# IL-6 Receptor Inhibition by Tocilizumab Attenuated Expression of C5a Receptor 1 and 2 in Non-ST-Elevation Myocardial Infarction

**DOI:** 10.3389/fimmu.2018.02035

**Published:** 2018-09-12

**Authors:** Hilde L. Orrem, Per H. Nilsson, Søren E. Pischke, Ola Kleveland, Arne Yndestad, Karin Ekholt, Jan K. Damås, Terje Espevik, Bjørn Bendz, Bente Halvorsen, Ida Gregersen, Rune Wiseth, Geir Ø. Andersen, Thor Ueland, Lars Gullestad, Pål Aukrust, Andreas Barratt-Due, Tom E. Mollnes

**Affiliations:** ^1^Department of Immunology, Oslo University Hospital, Rikshospitalet, Oslo, Norway; ^2^University of Oslo, Oslo, Norway; ^3^Division of Emergencies and Critical Care, Department of Anesthesiology, Oslo University Hospital, Rikshospitalet, Oslo, Norway; ^4^KG Jebsen Inflammation Research Centre, University of Oslo, Oslo, Norway; ^5^Linnaeus Centre for Biomaterials Chemistry, Linnaeus University, Kalmar, Sweden; ^6^Clinic of Cardiology, St. Olavs Hospital, Trondheim, Norway; ^7^Department of Circulation and Medical Imaging, Faculty of Medicine and Health Sciences, Norwegian University of Science and Technology, Trondheim, Norway; ^8^Research Institute of Internal Medicine, Oslo University Hospital, Rikshospitalet, Oslo, Norway; ^9^Faculty of Medicine, University of Oslo, Oslo, Norway; ^10^KG Jebsen Center for Cardiac Research, University of Oslo, Oslo, Norway; ^11^Center for Heart Failure Research, Oslo University Hospital, Oslo, Norway; ^12^Centre of Molecular Inflammation Research, Department of Clinical and Molecular Medicine, Norwegian University of Science and Technology, Trondheim, Norway; ^13^Department of Cardiology, Oslo University Hospital, Rikshospitalet, Oslo, Norway; ^14^Center for Clinical Heart Research, Oslo University Hospital, Ullevål, Oslo, Norway; ^15^Department of Cardiology, Oslo University Hospital, Ullevål, Oslo, Norway; ^16^Section of Clinical Immunology and Infectious Diseases, Oslo University Hospital, Oslo, Norway; ^17^Research Laboratory, Nordland Hospital, Bodø, Norway; ^18^K.G. Jebsen TREC, University of Tromsø, Tromsø, Norway

**Keywords:** complement, C5a receptors, C3a receptor, IL-6, myocardial infarction, inflammation

## Abstract

**Background:** Elevated interleukin-6 (IL-6) and complement activation are associated with detrimental effects of inflammation in coronary artery disease (CAD). The complement anaphylatoxins C5a and C3a interact with their receptors; the highly inflammatory C5aR1, and the C5aR2 and C3aR. We evaluated the effect of the IL-6 receptor (IL-6R)-antagonist tocilizumab on the expression of the anaphylatoxin receptors in whole blood from non-ST-elevation myocardial infarction (NSTEMI) patients. Separately, anaphylatoxin receptor expression in peripheral blood mononuclear cells (PBMC) from patients with different entities of CAD was investigated.

**Materials and Methods:** NSTEMI patients were randomized to one dose of tocilizumab (*n* = 28) or placebo (*n* = 32) and observed for 6 months. Whole blood samples drawn at inclusion, at day 2, 3 and after 6 months were used for mRNA isolation. Plasma was prepared for analysis of complement activation measured as sC5b-9 by ELISA. Furthermore, patients with different CAD entities comprising stable angina pectoris (SAP, *n* = 22), non-ST-elevation acute coronary syndrome (NSTE-ACS, *n* = 21) and ST-elevation myocardial infarction (STEMI, *n* = 20) were included. PBMC was isolated from blood samples obtained at admission to hospital and mRNA isolated. Anaphylatoxin-receptor-expression was analyzed with qPCR using mRNA from whole blood and PBMC, respectively.

**Results:** Our main findings were (i) Tocilizumab decreased C5aR1 and C5aR2 mRNA expression significantly (*p* < 0.001) and substantially (>50%) at day 2 and 3, whereas C3aR expression was unaffected. (ii) Tocilizumab did not affect complement activation. (iii) In analyzes of different CAD entities, C5aR1 expression was significantly increased in all CAD subgroups compared to controls with the highest level in the STEMI patients (*p* < 0.001). For C5aR2 and C3aR the expression compared to controls were more moderate with increased expression of C5aR2 in the STEMI group (*p* < 0.05) and C3aR in the NSTE-ACS group (*p* < 0.05).

**Conclusion:** Expression of C5aR1 and C5aR2 in whole blood was significantly attenuated by IL-6R-inhibition in NSTEMI patients. These receptors were significantly upregulated in PBMC CAD patients with particularly high levels of C5aR1 in STEMI patients.

## Introduction

Inflammation plays a pivotal role in the pathophysiology of coronary artery disease (CAD) from the establishment of the atherosclerotic plaque through rupture or erosion of the plaque leading to partial or total occlusion of the coronary vessel. This might lead to myocardial necrosis and thereby a myocardial infarction (MI). A total occlusion typically leads to ST-elevation in the electrocardiogram whereas a partial occlusion or an occlusion with collateral circulation doses not show these changes and are classified as unstable coronary syndromes. Unstable coronary syndromes with elevated levels of Troponin T, a marker of myocardial necrosis, are classified as non-ST-elevation MI (non-STEMI) whereas without rice in TnT are classified as non-ST-elevation acute coronary syndromes (1). Rapid restoration of coronary blood flow by re-opening of the occluded coronary vessel with percutaneous coronary intervention (PCI), has considerably improved outcome following MI. However, CAD is still associated with considerable morbidity and mortality (2).

Both the myocardial necrosis and the reperfusion of the infarcted myocardium activate inflammatory mechanisms. Innate and adaptive immune mechanisms are involved in this process and act together to orchestrate a response to damage (3). A balanced inflammatory response is required for proper healing following myocardial infarction (MI), whereas excessive inflammation could give rise to collateral tissue damage with detrimental effects on the myocardium (4). The complement system is an important sensor and effector system of innate immunity and plays a role in all phases of CAD (5). The complement system exerts its main inflammatory functions through proteolytic activation of C3 and C5, which upon cleavage liberate the complement anaphylatoxins C5a and C3a. The anaphylatoxins bind to their respective receptors: the C5a receptor 1 and 2 (C5aR1, C5aR2) and the C3a receptor (C3aR) (6), and the C5a-C5aR1-axis seems to be involved in atherogenesis and CAD (7–9). C5aR inherits an inflammatory role following tissue injury stimulating the release of cytokines like tumor necrosis factor (TNF), interleukin (IL)-1β, IL-6, and chemokines, e.g., IL-8 (10), and induce thrombogenicity by upregulation of tissue factor (11). The effect of activating C5aR2 and C3aR are more diverse and the effect of activating these receptors in the context of acute coronary syndromes (ACS) is at present less clear.

IL-6 and complement may both contribute to the progression of cardiovascular diseases (5, 12, 13) but there are limited data on the interaction between these inflammatory proteins. In a mouse sepsis model, IL-6 inhibition reduced the expression of tissue C5aR (14), but to the best of our knowledge the effects of IL-6 inhibition on the anaphylatoxin receptor expression in human CAD have not been investigated. In a recent study, the IL-6 receptor (IL-6R) antagonist tocilizumab reduced C-reactive protein (CRP) and percutaneous coronary intervention (PCI)-related troponin T (TnT) release in patients with non-ST-elevation myocardial infarction (NSTEMI) (15). In the present study, we aimed to investigate the expression of the anaphylatoxin receptors in a sub-group of this patient cohort (15). Additionally, anaphylatoxin receptor expression was investigated in samples from patients with different entities of CAD before any intervention was initiated.

## Materials and methods

In this study we included two different patient cohorts: one cohort consisting of NSTEMI-patients randomized to anti-inflammatory treatment with an IL-6R antagonist or placebo where blood was sampled from inclusion, before treatment and with repeated measurements, and another cohort consisting of patients with different entities of CAD where blood samples were drawn at hospital admission, before treatment was given.

### NSTEMI patients treated with tocilizumab

**Figure 1:**
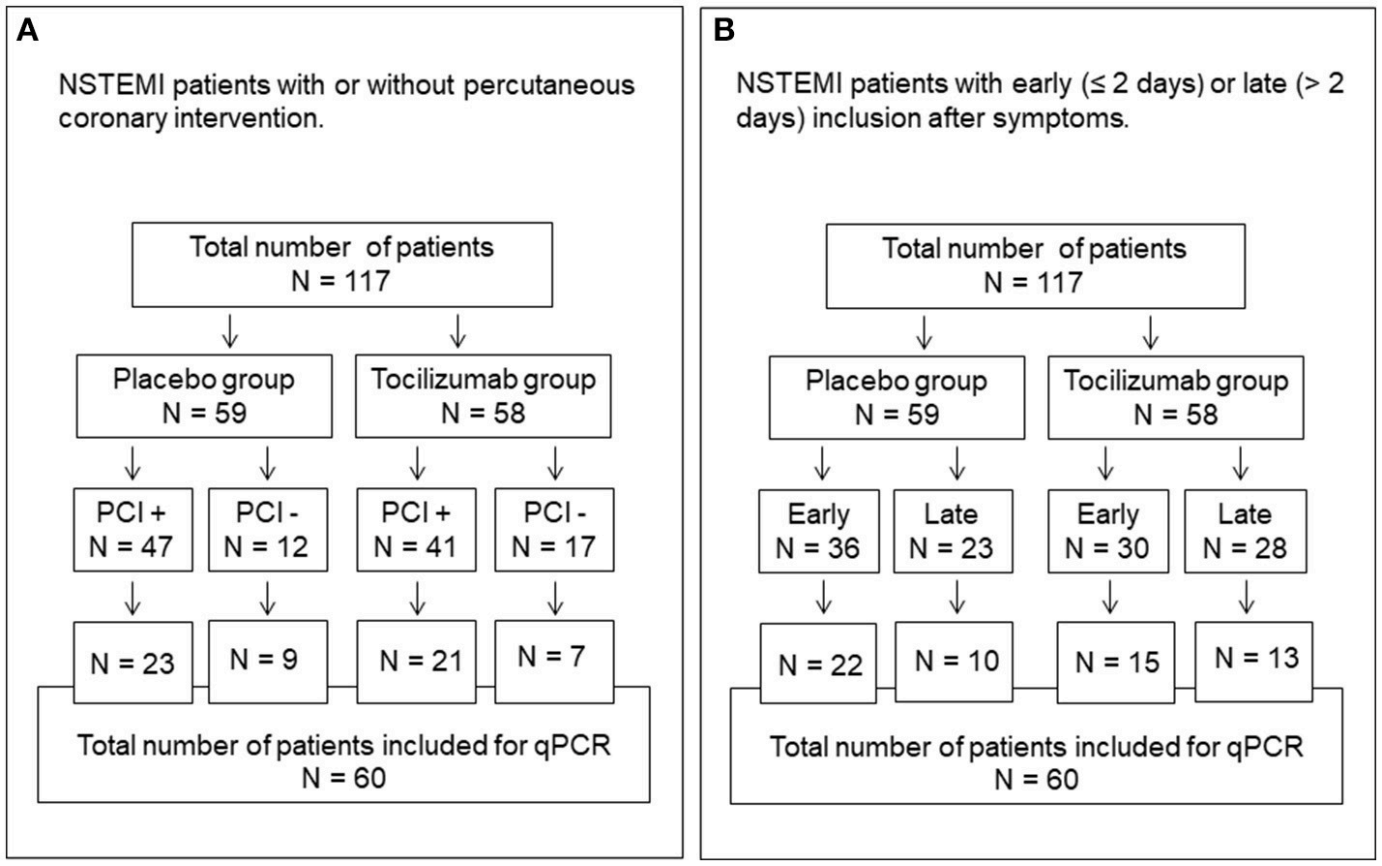
Flow chart showing the total number of patients and randomization to tocilizumab and placebo groups. **(A)** Number in each group with or without PCI. **(B)** Number in each group included early (≤2 days) or late (>2 days) from the onset of symptoms. NSTEMI, non-ST-elevated myocardial infarction.

The present work is a sub-study of a previously published double-blind, placebo-controlled two-center study on patients (*n* = 117) admitted with NSTEMI randomized to treatment with the IL-6R inhibitory monoclonal antibody tocilizumab (*n* = 58) or placebo (*n* = 59) (ClinicalTrails.gov, NCT01491074) (15). Tocilizumab was administrated as a single dose of 280 mg immediately prior to coronary angiography. This dose provides a complete IL-6 blockade for approximately 2 weeks (15). Briefly, patients between 18 and 80 years of age with NSTEMI scheduled for coronary angiography were included. Exclusion criteria were clinically significant cardiac disease other than CAD, disease or medication affecting inflammation, contraindications to the treatment drug and clinically unstable patients. Patients were included at a median of 2 days after symptom onset. There were no significant between-group differences in baseline characteristics (15). Fifteen age and sex-matched healthy controls were included. A flow chart describing the whole patient population randomized to tocilizumab or placebo and the number of patients with or without PCI, and with early (≤2 days) vs. late (>2 days) inclusion after symptoms onset is shown in Figure 1.

We evaluated the expression of anaphylatoxin receptors (C5aR1, C5aR2, and C3aR) in 60 of the patients treated with tocilizumab (*n* = 28) or placebo (*n* = 32). These patients represent all patients included at one of the two study centers (St. Olavs hospital). Due to lack of resources, we only investigated patients from half of the original study population. In this subgroup of patients, there was a significant difference in gender, but no other differences in baseline characteristics were found (Table 1). The whole study population (*n* = 117) was included for plasma complement activation analysis.

**Table 1:** Clinical and biochemical characteristics of the study population 1 (*n* = 60).

**Characteristics**	**Placebo**	**Tocilizumab**	***p*-value**
Total number, *n*	32	28	
Male sex, *n* (%)	32 (100)	23 (82)	**0.02**
Age, years, mean (SD)	59 (9)	58 (6)	1.00
Body mass index, kg/m^2^, median (IQR)	27 (25, 29)	28 (27, 30)	1.00
Hypertension, *n* (%)	10 (31)	15 (54)	0.12
Diabetes mellitus, *n* (%)	5 (16)	5 (18)	1.00
Previous myocardial infarction, *n* (%)	5 (16)	3 (11)	0.70
Current smoking, *n* (%)	13 (41)	9 (32)	0.60
Systolic blood pressure, BL, mmHg (SD)	135 (14)	138 (17)	1.00
Diastolic blood pressure, BL, mmHg (SD)	82 (11)	83 (10)	1.00
PCI, *n* (%)	23 (72)	21 (75)	1.00
LMWH BL, *n* (%)	29 (91)	26 (93)	1.00
CRP, BL, mg/L, median (IQR)	2.2 (0.7, 7.9)	3.3 (1.2, 6.7)	1.00
Troponin-T, BL, ng/L, median (IQR)	187 (87, 485)	107 (630)	1.00
Creatinine, BL, μmol/L, median (IQR)	77 (71, 90)	75 (63, 89)	1.00
Peak CRP, mg/L, median (IQR)	5.4 (1.2, 8.3)	5.4 (1.4, 18.7)	1.00
Peak Troponin-T, ng/L, median (IQR)	242 (92, 836)	128 (66, 937)	1.00
Peak Creatinine, μmol/L, median (IQR)	86 (74, 93)	81 (71, 95)	1.00

*Data are given as mean with (standard deviation, SD) or median with (25 and 75th percentile, IQR) and number with (%). BL, baseline values; PCI, percutaneous coronary intervention; LMWH, low molecular heparin administered before baseline; CRP, C-reactive protein; R, receptor. Bold value indicate statistical significance*.

### Patients with various CAD entities

Three patient groups with different entities of CAD, described in detail elsewhere (16), were examined with respect to anaphylatoxins receptor expression in blood samples obtained at admission to hospital. CAD was defined as coronary artery stenosis >50% verified by coronary angiography. The three patient entities were defined as: (i) stable angina pectoris (SAP) (*n* = 22), defined as episodes with reversible ischemic chest pain, referred to elective coronary angiography. (ii) Non-ST-elevation acute coronary syndromes (NSTE-ACS) that included unstable angina and NSTEMI patients (*n* = 21), defined as angina at rest or crescendo angina, referred to urgent coronary angiography within 48 h. (iii) STEMI (*n* = 20) defined as elevated plasma levels of Troponin T (TnT; at least one value above the 99th percentile) together with ischemic symptoms and ST-segment elevation or new left bundle branch block in the electrocardiogram referred to immediate coronary angiography and PCI if indicated (16). Patients that had malignant or chronic inflammatory diseases, intercurrent infections, or were treated with glucocorticosteroids were not included. Age and sex-matched healthy controls (*n* = 29) were also included.

### Blood sampling protocol

#### NSTEMI tocilizumab study

Blood samples drawn at the time of inclusion, i.e., before study medicine was given and angiography performed, at day 2 and 3 following inclusion and after 6 months were included in this sub-study. Blood was collected in EDTA vacutainer tubes (BD Biosciences, Plymouth, UK), kept on crushed ice and centrifuged within 30 min at 2,500 *g* for 20 min at 4°C. Plasma was stored at −80°C until analyzed, and samples were thawed only once. Whole blood (3 mL) was collected in Tempus Blood RNA tubes (ThermoFischer, Paisley, UK) from patients and healthy controls ensuring immediate lysis of all blood cells and stabilization of RNA. Tempus Blood RNA tubes were stored at −80°C until RNA preparation.

#### Patients with different CAD entities and healthy controls

Venous blood was drawn from healthy controls and patients with SAP and NSTE-ACS before angiography. Arterial blood was drawn from the arterial cannula immediately before coronary angiography in patients with STEMI. Peripheral blood mononuclear cells (PBMCs) were isolated from heparinized blood in all three patient groups and the healthy controls by Isopaque-Ficoll (Lymphoprep, FreseniusKabi Norge AS, Oslo, Norway) gradient centrifugation within 1 h after sampling, stored at −80°C as cell pellets until RNA isolation was performed.

### RNA isolation and quantitative PCR (qPCR)

#### NSTEMI tocilizumab study

Whole blood RNA purification was performed by Aaros Applied Biotechnology, Aarhus, Denmark. mRNA from the healthy controls was isolated using Tempus Spin RNA isolation Kit (ThermoFischer, Paisley, UK). cDNA was produced using the high capacity cDNA reverse transcriptase kit (Applied Biosystem, Foster City, CA). TaqMan qPCR primers (FAM-MGB dye-labeled) were purchased from Applied Biosystems for the following genes: C5aR1 (HS00704891), C5aR2 (Hs01933768) and C3aR (Hs0026963). Beta-2-microglobulin (HS 00187842) was stably expressed and used as endogenous control. Each sample was analyzed in triplicate and the reaction was run in 96 well-MicroAmp optical reaction plate on a StepOnePlus system (Applied Biosystems).

#### Patients with different CAD entities

RNA from PBMC was isolated using RNeasy Mini Kit (Qiagen, Hilden, Germany). cDNA was synthesized using qScript cDNA SuperMix (Qantabio, Beverly MA). SybrGreen primers were used for qPCR (primer sequences can be given upon request) with GAPDH as endogenous control. Each sample was analyzed in duplicate in 384 well-optical reaction plate on a 7900 HT Fast Real-time PCR system.

### Complement activation

Plasma complement activation was evaluated by quantification of the terminal complement complex (TCC) in its soluble form (sC5b-9) using an enzyme-linked immunosorbent assay (ELISA) previously described in detail (17). Briefly, the mAb, aE11, which binds to a neoepitope exposed in C9 when incorporated into the C5b-9 complex, was used as capturing antibody and a biotinylated monoclonal anti-C6 (clone 9C4) was used for detection. The level was related to the International Complement Standard #2, defined to contain 1,000 complement arbitrary units (CAU) per mL (17).

### Data presentation and statistical analysis

Statistical analysis was performed with IBM SPSS Statistics 24 (Armonk, NY) or Graph Pad Prism, version 7 (San Diego, CA). Differences between two groups were tested with *t*-test or Mann-Whitney *U* test when the data were not normally distributed. Differences between more than two groups were tested with ordinary one-way ANOVA or with Kruskal-Wallis test dependent on distribution. Change from baseline was calculated for each time point (e.g., time point-baseline). Longitudinal data were analyzed with Friedman test followed by Wilcoxon signed-rank test to compare the specific time point with baseline levels within each treatment group. To compare differences in categorical data between groups the Chi-square test was used. Correlation analysis was measured by the Spearman correlation test. Bonferroni correction was used to correct for multiple testing. Results are given as median with interquartile range or mean with 95% confidence interval (CI). All tests were two-sided and a *p*-level of < 0.05 was regarded as statistically significant.

### Ethics

Both studies were approved by the Regional Committee for Medical and Health Research Ethics of South-Eastern Norway and the tocilizumab study also by The Norwegian Medicine Agency and both studies were conducted in accordance with the Declaration of Helsinki. All participants provided written informed consent.

## Results

### The effect of IL-6R inhibition on anaphylatoxin receptor expression in whole blood from NSTEMI patients

#### C5aR1

**Figure 2:**
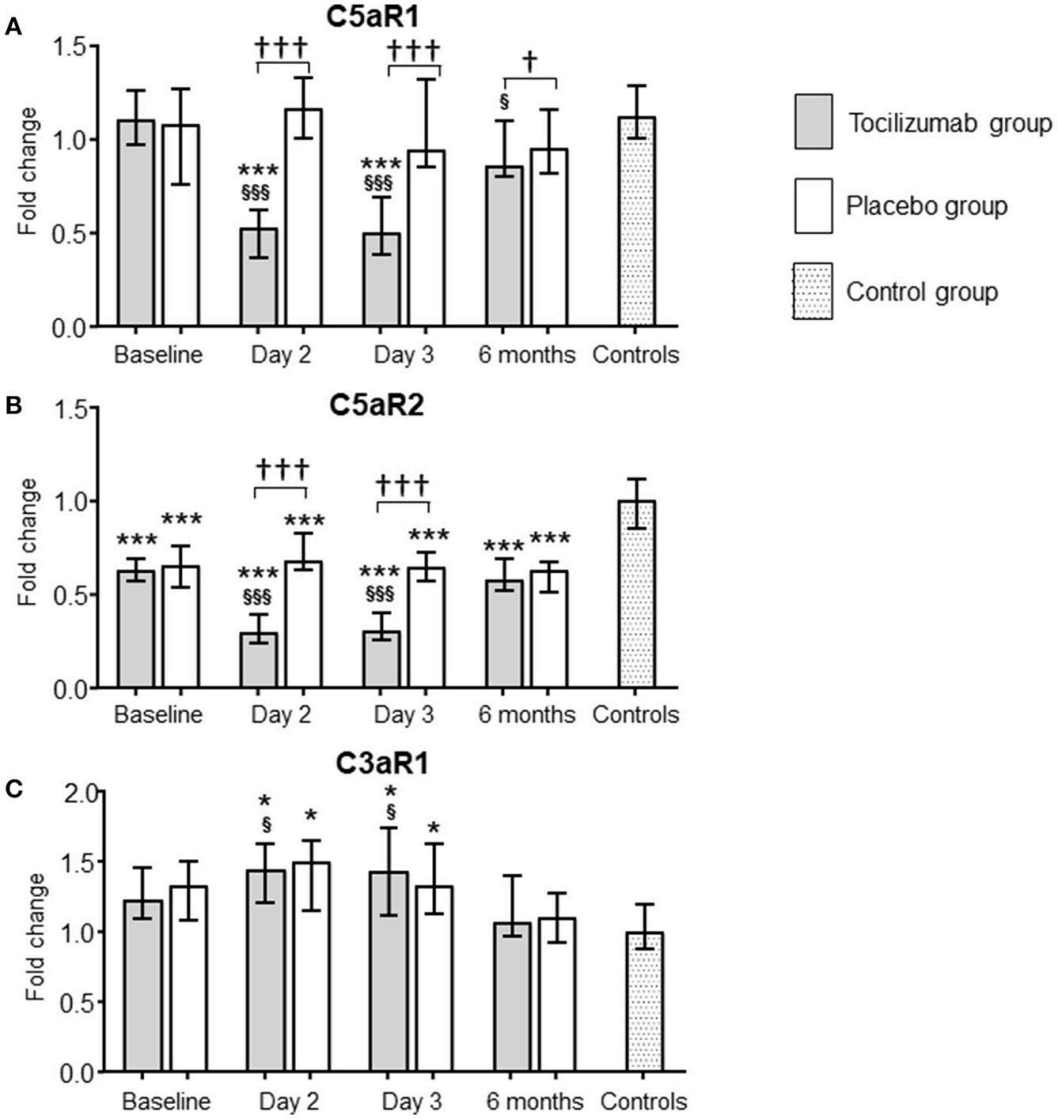
Effect of tocilizumab on the expression of C5aR1, C5aR2, and C3aR in NSTEMI patients. The effect of tocilizumab, a monoclonal antibody inhibiting interleukin 6 receptor (IL-6R), on the expression of the three complement anaphylatoxin receptors [C5aR1 **(A)**, C5aR2 **(B)**, and C3aR **(C)**] was investigated in patients with non-ST-elevation myocardial infarction (NSTEMI). mRNA levels were quantified by qPCR and related to the reference gene beta-2-microglobulin. The tocilizumab group (gray bars, *n* = 28) and the placebo group (white bars, *n* = 32) are presented at four different time-points. Baseline levels show the receptor expression at inclusion, i.e., after hospital admission, before treatment was given. Follow-up time points were day 2 and 3, and 6 months. A group of healthy individuals (*n* = 15) were included as controls. The qPCR results were quantified using the 2^−ΔΔCT^ method, normalized to reference genes and presented as fold change with the healthy controls as calibrator. Data are given as median and 95% CI. *P < 0.05, ***P < 0.001 vs. healthy controls. ^†^*P* < 0.05, ^†††^*P* < 0.001 differences in change from baseline between tocilizumab and placebo. ^§^*p* < 0.05, ^§§§^*p* < 0.001 vs. baseline.

Expression of C5aR1 was significantly lower in the tocilizumab group compared to the placebo group at day 2 and 3 (Figure 2A). Compared to baseline and the healthy controls, the expression of C5aR1 at day 2 and 3 was significantly lower in the tocilizumab group, whereas no difference was observed for the placebo group. After 6 months the expression of C5aR1 in the tocilizumab group was still significantly lower compared to baseline, which was not the case for the placebo group. Compared to healthy controls there was no difference at baseline or after 6 months in any of the two patient groups (Figure 2A).

#### C5aR2

Expression of C5aR2 was significantly lower in the tocilizumab group compared to the placebo group at day 2 and 3 (Figure 2B). Compared to baseline levels, the expression of C5aR2 was significantly lower in the tocilizumab group at day 2 and 3, whereas no such difference was observed in the placebo group. There were no differences between the two patients groups at baseline or after 6 months. Compared to healthy controls, C5aR2 expression was significantly decreased in the tocilizumab group and the placebo group during the whole study period (Figure 2B).

#### C3aR

C3aR expression behaved strictly different from the C5a receptors. There were no differences in receptor expression between the tocilizumab group and the placebo group at any of the time points (Figure 2C). In the tocilizumab group there was a significantly higher expression of C3aR at day 2 and 3 when compared to baseline, whereas both patients groups had significantly higher levels at day 2 and 3 compared to healthy controls (Figure 2C). At baseline and after 6 months, there were no differences in C3aR expression between the patient groups and health controls (Figure 2C).

### Effects of coronary intervention and time from symptom onset to inclusion on the expression of anaphylatoxin receptors

**Figure 3:**
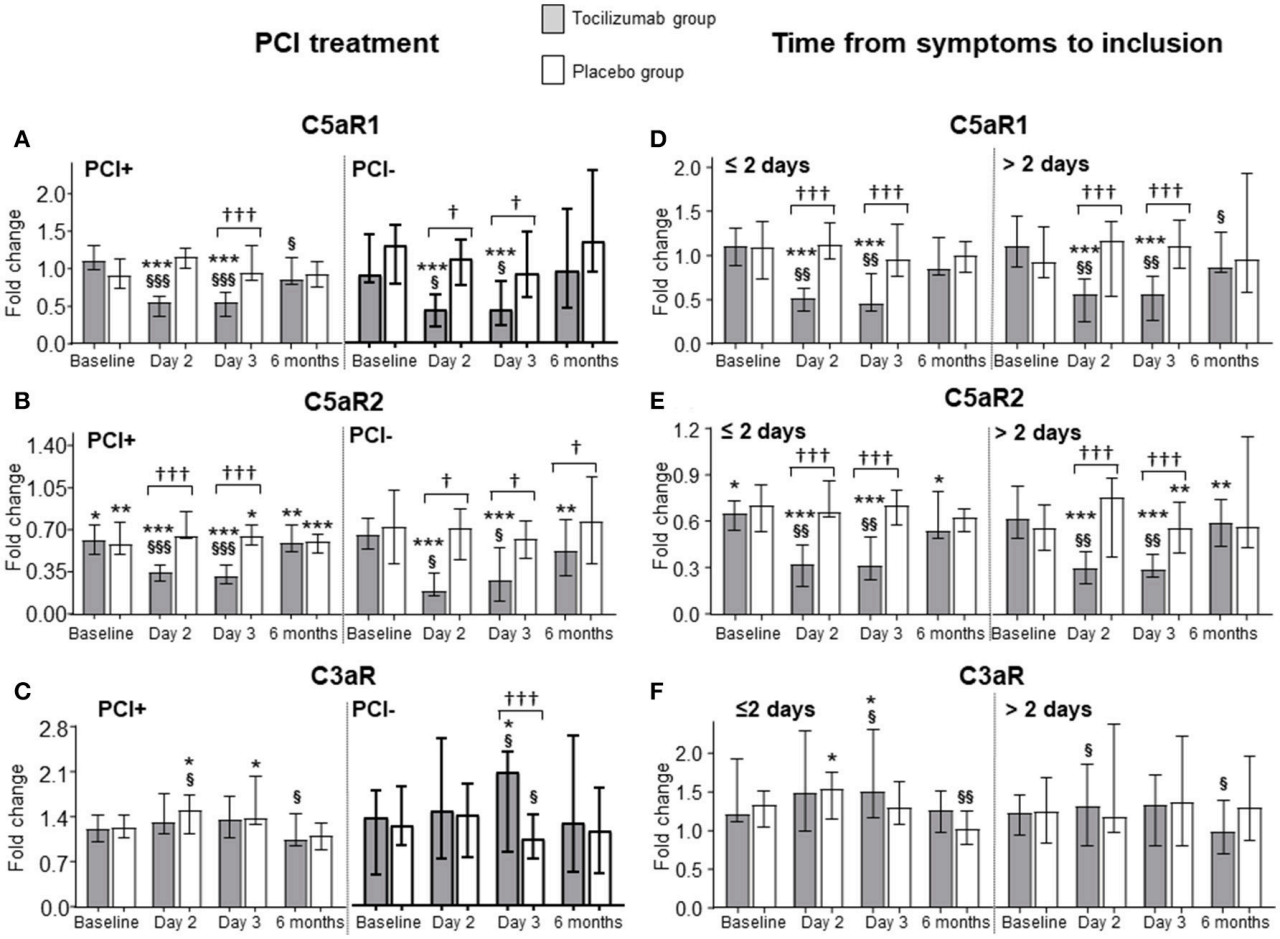
Effect of coronary intervention and time of inclusion on the expression of C5aR1, C5aR2, and C3aR in NSTEMI patients. Expression level of complement anaphylatoxin receptors C5aR1 **(A,D)**, C5aR2 **(B,E)**, and C3aR **(C,F)** in patients with non-ST-elevation myocardial infarction (NSTEMI) receiving placebo (*n* = 32) or tocilizumab (*n* = 28) divided into two groups according to percutaneous coronary intervention (PCI) (23 placebo and 21 tocilizumab, gray bars) or not (7 placebo and 9 tocilizumab, white bars) **(A–C)**, and divided into two groups according to inclusion ≤2 days (22 placebo and 15 tocilizumab, gray bars) or >2 days (10 placebo and 13 tocilizumab, white bars) from symptom onset **(D–F)**. Baseline levels show the receptor expression at inclusion, i.e., after hospital admission, before treatment was given. Follow-up time points were day 2 and 3, and 6 months. A group of healthy individuals (*n* = 15) were included as controls. The qPCR results were quantified using the 2^−ΔΔCT^ method, normalized to reference genes and presented as fold change with the healthy controls as calibrator. Data are given as median and 95% CI. *P < 0.05, **P < 0.01, ***P < 0.001 vs. healthy controls. ^†^*P* < 0.05, ^†††^*P* < 0.001 differences in change from baseline between tocilizumab and placebo. ^§^*p* < 0.05, ^§§^*p* < 0.01, ^§§§^*p* < 0.001 vs. baseline.

The effect of tocilizumab could potentially depend on whether the patients were treated with PCI or not, or whether they were included early (≤2 days) or late (>2 days) from the onset of symptoms. However, the pattern of the C5aR1, C5aR2 and C3aR expression was virtually identical in patients with or without PCI (Figures 3A–C) and in patients included early or late (Figures 3D–F). A flow chart of the patients is shown in Figure 1.

### Systemic complement activation in NSTEMI patients

**Figure 4:**
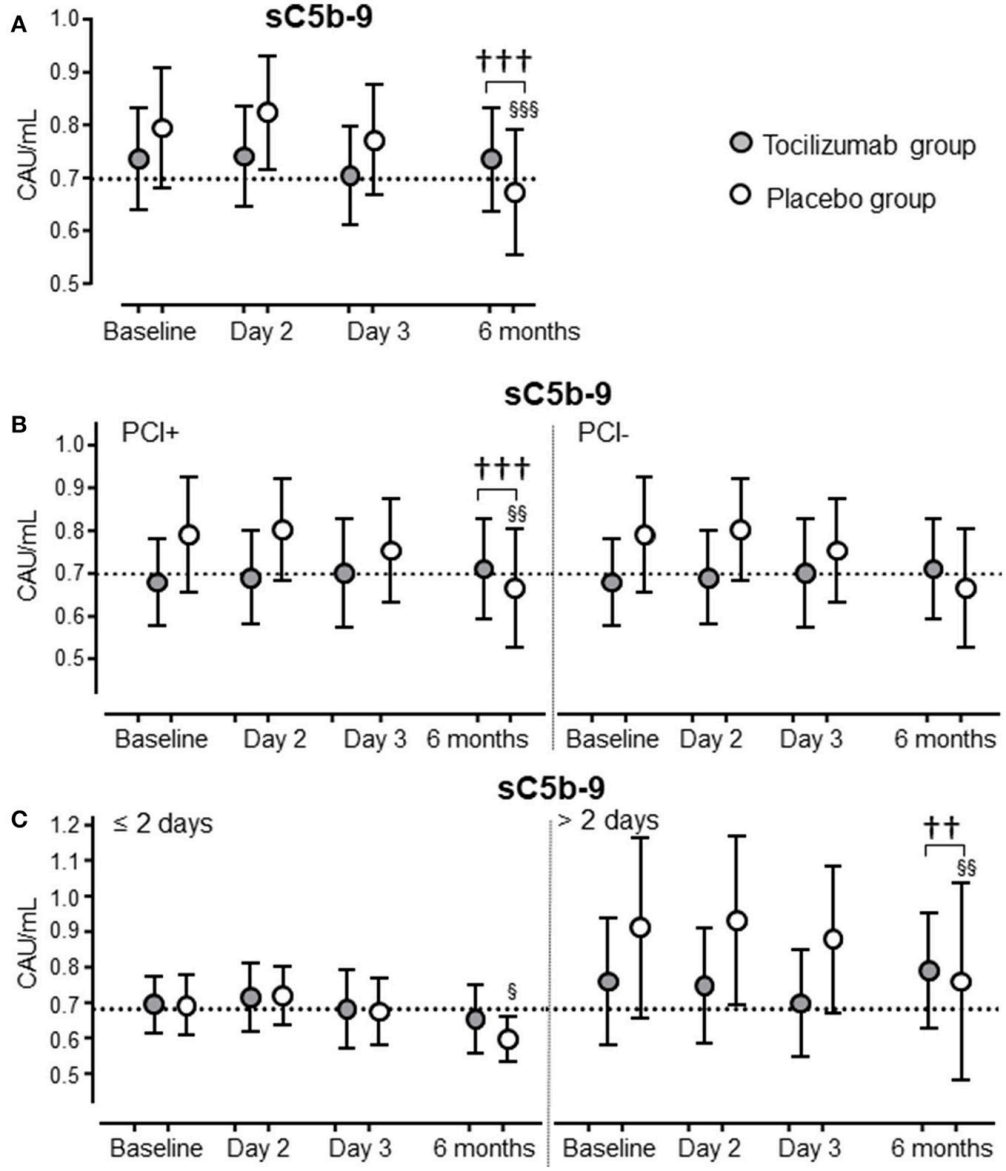
Systemic complement activation in NSTEMI patients treated with tocilizumab or PCI. Complement activation, as evaluated by plasma sC5b-9, is shown for NSTEMI patients (total *n* = 117) treated with tocilizumab (*n* = 58; closed circles) or placebo (*n* = 59; open circles) **(A)**. Complement activation, as evaluated by plasma sC5b-9, is shown for the same population of NSTEMI patients further divided into two groups according to percutaneous coronary intervention (47 placebo and 41 tocilizumab) or not (12 placebo and 17 tocilizumab) **(B)**, and according to inclusion ≤2 days (36 placebo and 30 tocilizumab) or >2 days (23 placebo and 28 tocilizumab) from symptom onset to inclusion **(C)**. The dotted line represents normal upper level of sC5b-9 in healthy blood donors. Data are given as mean and 95% CI.*P* < 0.01,*P* < 0.001 differences in change from baseline between tocilizumab and placebo. ^§^*p* < 0.05, ^§§^*p* < 0.01, ^§§§^*p* < 0.001 vs. baseline.

To see if inhibition of IL-6R affected complement activation, sC5b-9 was evaluated in all patients in the tocilizumab study (*n* = 117) from baseline to day 3, and at 6 months follow up. Plasma concentration of sC5b-9 did not change over time in the NSTEMI patients (Figure 4A). Tocilizumab had no effect on the degree of systemic complement activation. The same pattern was seen regardless of PCI treatment or not (Figure 4B) and independent of early (≤2 days) or late (>2 days) inclusion from the onset of symptoms (Figure 4C).

### Association between the expression of anaphylatoxin receptors and key biomarkers in the NSTEMI patients during hospitalization

The original tocilizumab study found a fall in leukocytes in the tocilizumab-group, primarily caused by a decrease in neutrophils from baseline to day 3 which led to a significant between-group difference in change from baseline (15). The same statistical differences were also found in the sub-group of patients studied here (Table 2). We found no correlation between change in neutrophils and change in expression level of any of the three anaphylatoxin receptors in the treatment group (Supplementary Table 1). However, in the placebo group there was a significant correlation between change in neutrophils and change in the expression level of C5aR1 and C5aR2 (Supplementary Table 1).

**Table 2:** Baseline values and values at day 3 for different relevant biomarkers in the NSTEMI patients.

	**Group**	**Baseline**	**Day 3**	
IL-6 pg/mL	Placebo	3.0 (1.2–5.1)	3.2 (1.7–6.3)	^†^
	Tocilizumab	2.4 (1.3–4.5)	22(14.3–29)^***^	
sIL-6R ng/mL	Placebo	71 (13)	70 (11)	^†††^
	Tocilizumab	64 (11)	88 (8.4)	
Leukocytes (10^9^/L)	Placebo	7.6 (1.7)	7.6 (1.3)	^†††^
	Tocilizumab	8.0 (2.4)	4.9 (1.6)	
Neutrophils (10^9^/L)	Placebo	4.5 (3.3–5.7)	4.3 (3.7–5.2)	^†††^
	Tocilizumab	5.1 (3.2–6.0)	1.9 (1.2–2.7)	
Monocytes (10^9^/L)	Placebo	0.7 (0.2)	0.7 (0.6–0.9)	
	Tocilizumab	0.7 (0.5–0.9)	0.6 (0.2)	
Lymphocytes (10^9^/L)	Placebo	2.0 (0.5)	2.1 (0.6)	
	Tocilizumab	2.0 (0.7)	1.9 (1.6–2.5)	

*Data are given as mean with (standard deviation, SD) or median with (25 and 75th percentile, IQR). IL, interleukin; R, receptor; s, soluble*.

*** *p < 0.001 comparing differences within group from baseline*.

† *p < 0.05*,

††† *p < 0.001 comparing between-group differences in change from baseline*.

In the original tocilizumab study, IL-6 and sIL-6R increased significantly from baseline to day 3 in the tocilizumab-treated patients (15). A similar pattern was found in the sub-group of patients investigated in this study (Table 2). In the tocilizumab group no correlation was found between change in expression for any of the anaphylatoxin receptors and IL-6 in the tocilizumab. sIL-6R correlated with the expression of C3aR in the tocilizumab-treated patients (Supplementary Table 1). In the placebo group we found a significant correlation between all three anaphylatoxin receptors and IL6 whereas no correlation was found for sIL-6R (Supplementary Table 1).

### Associations between the anaphylatoxin receptors and CRP and TnT in the NSTEMI patients during hospitalization

We evaluated whether AUC for the three anaphylatoxin receptors showed any correlation with AUC for CRP and TnT representing the primary and most important secondary endpoint, respectively, in the original tocilizumab study (15) (Table 3). There was a significant correlation between C5aR1 and C3aR, but not C5aR2, and CRP in the placebo group, whereas only C3aR was correlated with CRP in the tocilizumab group (Table 3; Supplementary Figure 1). TnT correlated significantly with all three receptors in the placebo group, whereas only C5aR1 correlated with TnT in the tocilizumab group (Table 3; Supplementary Figure 1). All correlations between receptor expression and CRP and TnT were positive. The regression plots, however, show negative regression lines since the statistics were calculated on delta-CT values and a decrease in the delta-CT value represents an increase in receptor expression.

**Table 3:** Spearman Rho correlation between AUC during hospitalization for CRP and TnT and the three anaphylatoxin receptors in the NSTEMI patients.

		**AUC**	**AUC**	**AUC**
		**C5aR1**	**C5aR2**	**C3aR**
AUC CRP	Placebo	0.431^*^	0.214	0.506^**^
	Tocilizumab	0.264	0.082	0.338
AUC TnT	Placebo	0.399^*^	0.494^**^	0.400^*^
	Tocilizumab	0.534^*^	0.315	0.259

*AUC, area under the curve; TnT, troponin T; CRP, C-reactive protein; R, receptor*.

Data: Spearman Rho correlation coefficient with

* *p < 0.05*,

** *p < 0.01*.

*Bold values indicate statistical significance*.

### Expression of anaphylatoxin receptors in PBMC from patients with different CAD entities

In order to explore whether the expression of anaphylatoxin receptors is dependent on the severity of CAD, independent on any intervention, we investigated the expression of these receptors in PBMCs in samples obtained from patients admitted to hospital comprising three different entities of CAD: SAP (*n* = 22), NSTE-ACS (*n* = 21), and STEMI (*n* = 20).

**Figure 5:**
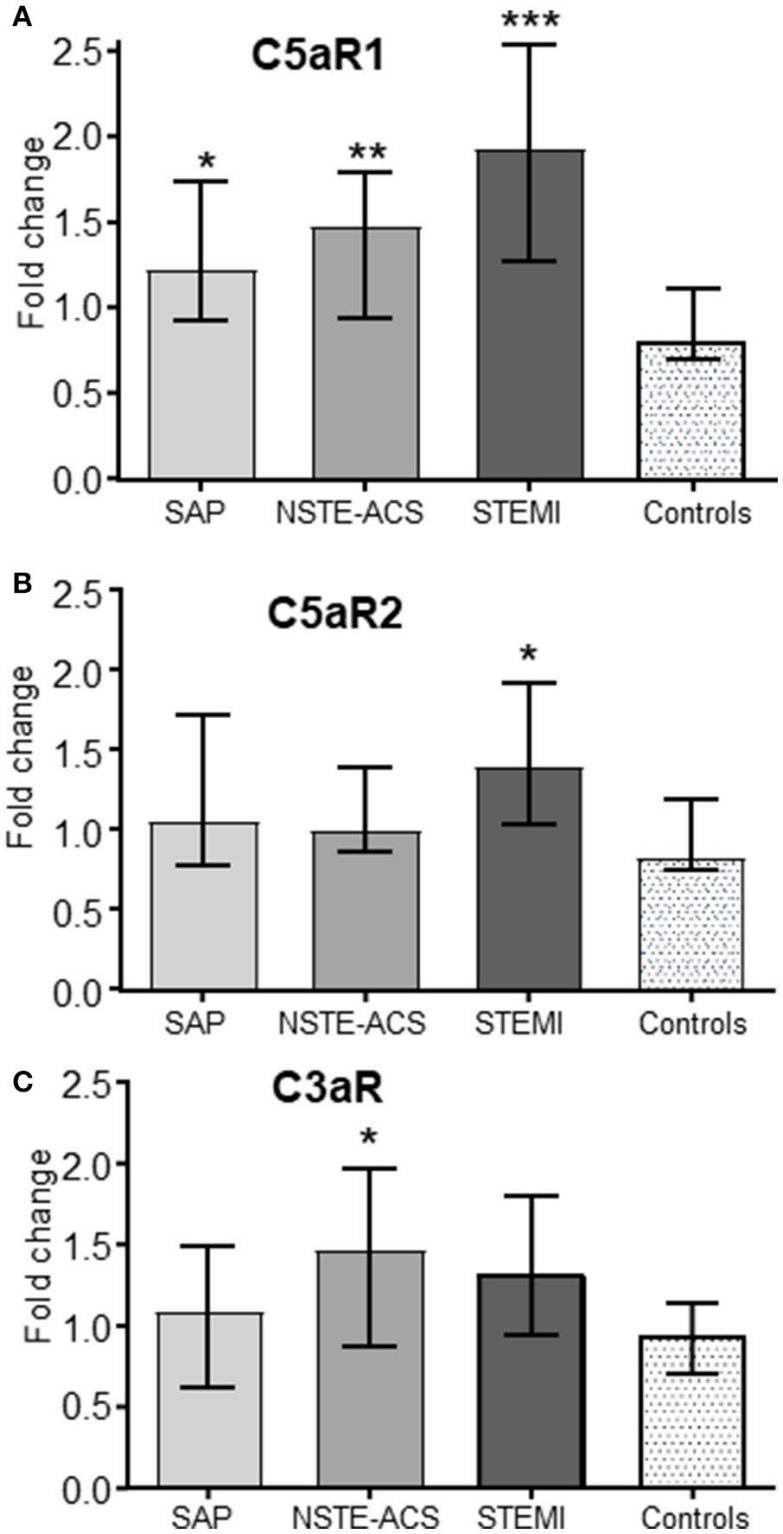
Expression of C5aR1, C5aR2 and C3aR in patients with various entities of CAD. Expression of the C5aR1 **(A)**, C5aR2 **(B)**, and C3aR **(C)** in three different patients groups with verified coronary artery disease (CAD): stable angina pectoris (SAP, *n* = 22), non-ST-elevation acute coronary syndromes (NSTE-ACS, *n* = 21) and ST-elevation myocardial infarction (STEMI, *n* = 20). A group of healthy age and sex-matched individuals were included as controls (Ctrls, *n* = 29). mRNA levels were quantified by qPCR using the 2^−ΔΔCT^ method, normalized to reference genes (GAPDH) and presented as fold change with the healthy controls as calibrator. Data are given as median and 95% CI. Statistical significant differences are indicated between the patient populations and the healthy controls. *p < 0.05, **p < 0.01, ***p < 0.001 vs. healthy controls.

Whereas C5aR1 expression was significantly increased in all CAD subgroups compared to healthy controls with the highest levels in the STEMI patients (Figure 5A), the increase in C5aR2 and C3aR were more moderate, showing significantly increased levels as compared with controls in the STEMI group (C5aR2) and NSTE-ACS group (C3aR) only (Figures 5B,C).

## Discussion

This study demonstrates for the first time that inhibiting IL-6R profoundly attenuated the expression of C5aR1 and C5aR2 in peripheral whole blood in NSTEMI patients. Treatment with PCI is known to cause a reperfusion injury, which in itself can enhance inflammation. However, the effect on the anaphylatoxin receptor expression seen in this study was independent of treatment with PCI or time between debut of symptoms and inclusion. In contrast, C3aR expression was not affected by the IL-6-inhibitory treatment. Moreover, changes in C5aR1 was significantly correlated with changes in TnT during tocilizumab treatment suggesting the beneficial effect of IL-6R inhibition at least partly could involve downregulation of the inflammatory C5aR1.

Inflammation plays a pivotal role in the wake of a MI being essential for cardiac repair (18). However, sustained and excessive inflammation may contribute to increased tissue damage and is associated with worse prognosis in ACS (19). Elevated levels of inflammatory markers like CRP, IL-6 and C5a are related to the detrimental effects of inflammation in CAD (5, 13, 19–22) and anti-inflammatory treatment is suggested to improve outcome after MI (23). Genetic studies suggest that inhibiting either IL-6 or complement could prove beneficial in patients with CAD (24–26), and it has recently been shown that a single dose of tocilizumab attenuates the increase in CRP and PCI-related TnT release in NSTEMI patients (15).

The activation of C5aR1 induces pro-inflammatory effects like recruitment and activation of inflammatory cells and enhanced cytokine and chemokine production. Experimental studies have shown reduction in infarct size and inflammation when the C5a/C5aR1-axis has been attenuated (27–30). Furthermore, lack of C5aR1 on circulating leukocytes led to reduced infarct size and improved clinical outcome in an *in vivo* mouse model of MI (31). IL-6 inhibition is previously shown to attenuate expression of anaphylatoxin receptors in an experimental model of sepsis (14). Herein, we show a similar pattern in NSTEMI patients with a significant downregulation of C5Ra1 by tocilizumab in the first days following NSTEMI. Notably, this downregulation was significantly correlated with TnT release in the tocilizumab group suggesting that downregulation of C5aR1 might contribute to the attenuated TnT release by tocilizumab seen in these patients (15). The gradual increase in C5aR1 expression in the different CAD subgroups from SAP through NSTE-ACS with the highest level in STEMI patients may further support a role for this receptor in plaque progression and destabilization.

C5aR2, previously considered a non-signaling receptor, has been shown to have both pro- and anti-inflammatory effects and its function seems to be dependent on cell type, disease context and species (32). In experimental studies of CAD, there is some evidence that antagonizing C5aR2 might have beneficial effects (9). In the present study we showed a downregulation of C5aR2 by tocilizumab in NSTEMI patients. However, changes in C5aR2 were not correlated with changes in TnT during tocilizumab treatment, and in contrast to C5aR1 expression, the changes in C5aR2 expression in PBMC in the different CAD subgroups were rather modest.

In the NSTEMI patient group, a reduction in C5aR2 expression was observed both in the tocilizumab group and the placebo group, throughout the whole study period. The expression of C5aR2 is known to be attenuated in the context of inflammation (33) and the reduced level of C5aR2 in both the placebo group and the tocilizumab group even observed at inclusion might be due to the inflammatory response caused by the MI itself. We did not find the same reduction in C5aR2 expression compared to controls in the PBMC CAD group. The reason for this is unknown, but might be due to differences in time of sampling in relation to the myocardial injury, different expression level in different cell types or different methods. The effect on C5aR2 seen after 6 months might be related to the enhanced inflammation caused by the reperfusion injury caused by treatment with PCI. Also attenuating IL-6, which is a pleiotrop cytokine, might indirectly change the expression of C5aR2. Thus, the effects of C5aR2 in the setting of CAD and myocardial damage are still unclear and needs further investigations.

C3aR was previously regarded as a pro-inflammatory receptor but recent studies support a more complex effector function for this receptor with anti-inflammatory effects in the acute phase of inflammation by preventing neutrophil mobilization from the bone marrow (34). In an experimental study of intestinal ischemia and reperfusion injury, C3aR was shown to ameliorate ischemia-reperfusion injury in mice (35). Herein, we found a marked increase in C3aR expression in NSTEMI patients that was not modulated by tocilizumab. Moreover, C3aR, but not the two C5a receptors, correlated positively with changes in CRP during IL-6 receptor inhibition. Whatever the effect of C3aR, these findings suggest that IL-6 differently affect the expression of the C5a receptors and C3aR.

There was a reduction in the number of leukocytes and particularly neutrophils in the tocilizumab-treated NSTEMI patients as demented in the original study (15). This could, however, not explain the decreased C5aR1 and C5aR2 expression. First, there was no correlation between the change in receptor expression and change in neutrophil levels in the tocilizumab group. Second, the amount of mRNA in all samples was identical coming mainly from granulocytes, lymphocytes and monocytes, which constitute the main amount of nucleated cells in peripheral blood. Also lymphocytes and monocytes express anaphylatoxin receptors. Lymphocytes have previously been found to express C5aR1 (36–39) and the two C5a-anaphylatoxin receptors are typically co-expressed (33). Monocytes also express all three anaphylatoxin receptors shown for the CAD-population in this study. Third, the decrease was explicitly seen for the C5a receptors and not for the C3aR, indicating that the decrease was selective. Taken together this supports a real reduction in expression of C5aR1 and C5aR2.

In the present study, we used whole blood and PBMC, precluding us for detecting individual cell populations as would have been possible using cell sorting. There is, however, an advantage of using whole blood for this purpose, since the cells are less manipulated and *in vitro* changes in cell activity is reduced and the changes are to a greater extent reflecting the *in vivo* situation.

No correlation between IL-6 or sIL-6R and the three different anaphylatoxin receptors in the tocilizumab-treated patients, were observed. Tocilizumab was administrated in doses high enough to give a total IL-6 blockade for about 2 weeks (15) thus the level of IL-6 or sIL-6R is rather irrelevant since the effect of the cytokine is totally blocked in all patients during the hospital stay. We did find a correlation between the anaphylatoxin receptors and IL-6 and sIL-6R in the in the placebo group consistent with rather little change in both IL-6 and the anaphylatoxin receptors during the time course in this group.

sC5b-9 did not increase in the present study which most likely was due to the relatively small MIs in the NSTEMI patients. Complement is however constantly activated at a low level and acts in the circulation as a humoral alarm system ready to respond to any danger threatening the host (40). Importantly, the absence of significant systemic complement activation does not preclude the presence of local activation with the ability to act at the site of damage. Thus, downregulation of the receptors for C5a might have beneficial effects both locally and systemically.

The present study has some limitations. The number of patients was rather low. Also, the lack of protein data on the anaphylatoxin receptor expression may weaken our conclusions. Finally, it should be emphasized that correlations do not necessarily mean any causal relationship and more mechanistic studies are needed to further explore the role of anaphylatoxin receptors in CAD.

In conclusion, a substantial and statistically highly significant reduction of C5a receptors was observed in NSTEMI patients treated with tocilizumab, and as for C5aR1, the downregulation correlated with attenuated TnT release. C5aR1 expression in PBMC did also reflect disease severity in another separate CAD population. The cross-talk between complement C5aR1 and IL-6 might contribute to the attenuated TnT release during tocilizumab treatment in these NSTEMI patients.

## Author contributions

HO, TM, PN, AB-D, PA, BH, OK, JD, BB, RW, LG, AY, TE, and SP contributed to conception and design; OK, LG, GA, BH, HO, IG, and KE contributed with acquisition of data; HO, TM, AB-D, PN, OK, GA, PA, BH, IG, KE, SP, AY, and TU contributed with analysis and interpretation of data; HO, TM, PN, AB-D, OK, and PA drafted the article; All authors critically revised the article and approved the final version.

### Conflict of interest statement

The authors declare that the research was conducted in the absence of any commercial or financial relationships that could be construed as a potential conflict of interest. The reviewer RR declared a past co-authorship with one of the authors TM to the handling editor.
