# Supplementary figures and images for: IL-6 Receptor Inhibition by Tocilizumab Attenuated Expression of C5a Receptor 1 and 2 in Non-ST-Elevation Myocardial Infarction
Source: Front Immunol. 2018 Sep 12;9:2035. doi: 10.3389/fimmu.2018.02035 (PMC6143659; doi:10.3389/fimmu.2018.02035)

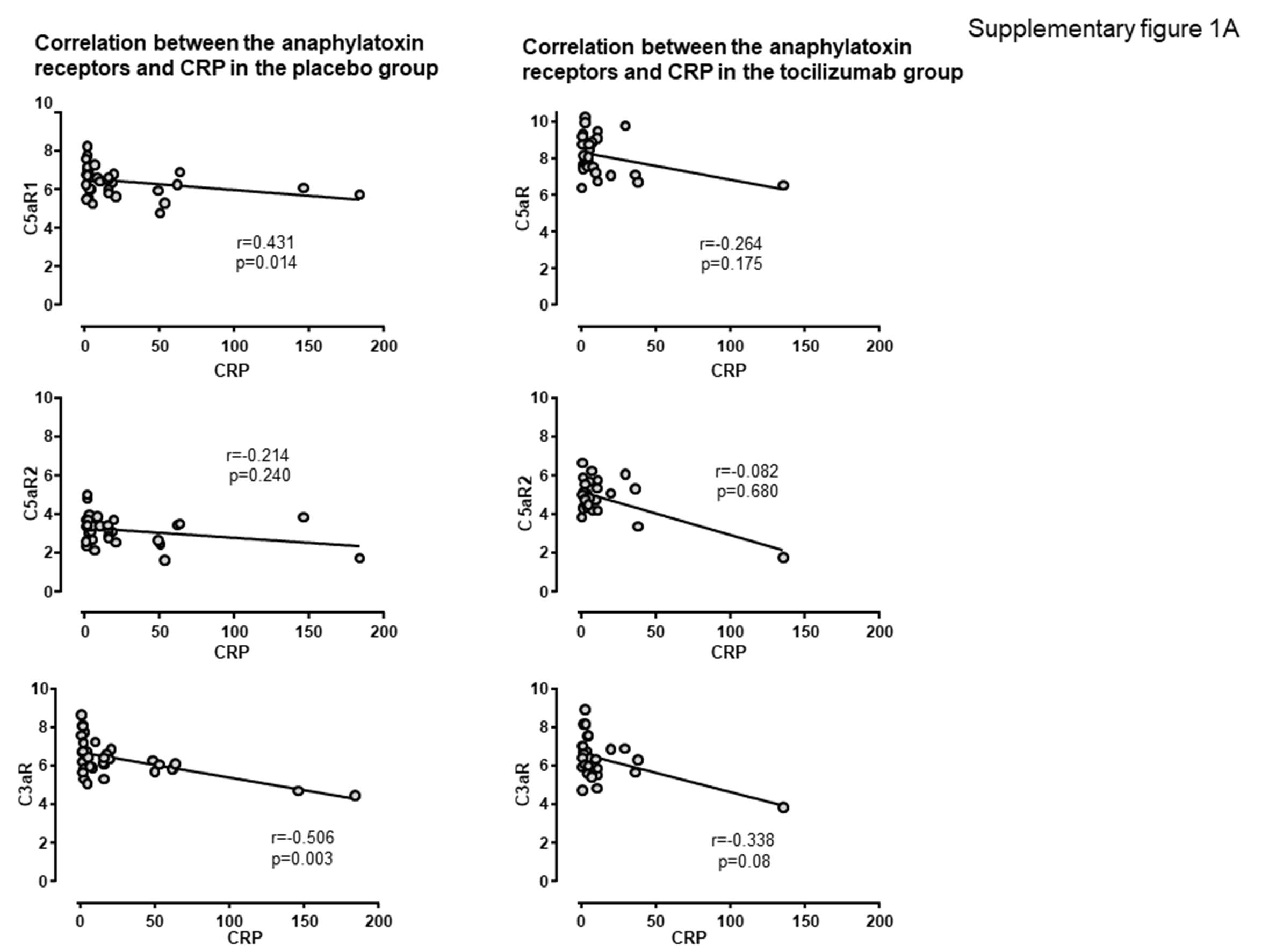

Supplement: Supplementary file 1 [file Image_1.TIF]

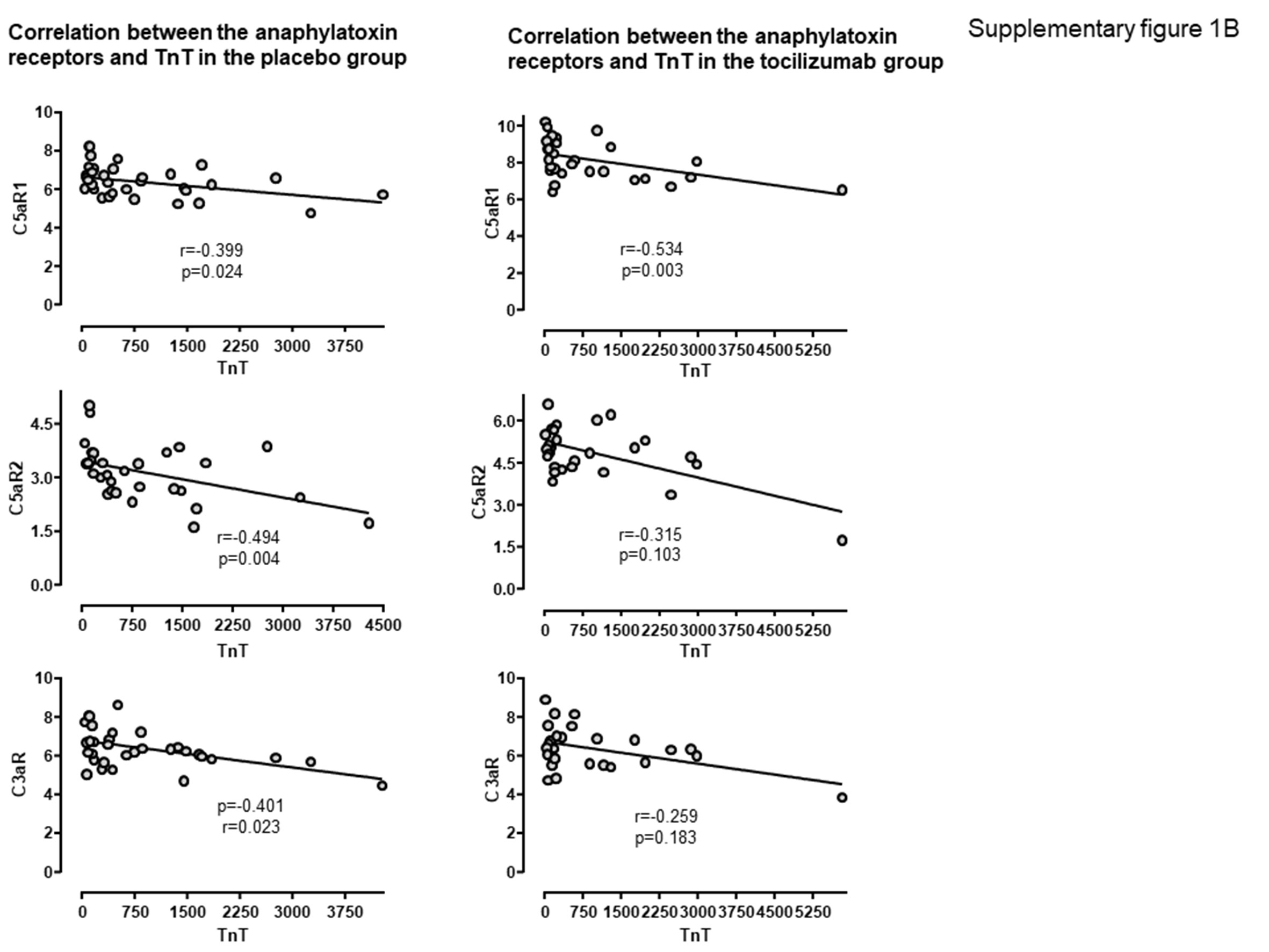

Supplement: Supplementary file 2 [file Image_2.TIF]
