# Supplementary material for: IL-6 Receptor Inhibition by Tocilizumab Attenuated Expression of C5a Receptor 1 and 2 in Non-ST-Elevation Myocardial Infarction
Source: Front Immunol. 2018 Sep 12;9:2035. doi: 10.3389/fimmu.2018.02035 (PMC6143659; doi:10.3389/fimmu.2018.02035)
Supplement: Supplementary file 3 [file Table_1.DOCX]

**Supplementary Table 1:** Spearman Rho for correlation between change in expression level of complement anaphylatoxin receptors in full blood and change in various key biomarkers in the tocilizumab-treated NSTEMI patients.

|  |  | **∆C5aR1** | **∆C5aR2** | **∆C3aR** |
| --- | --- | --- | --- | --- |
|  |  |  |  |  |
|  |  |  |  |  |
| ∆ IL-6 | Placebo | **0.646***** | **0.513**** | **0.674***** |
|  | Treatment | -0.008 | 0.049 | 0.093 |
|  |  |  |  |  |
| ∆s IL-6R | Placebo | -0.089 | -0.076 | -0.261 |
|  | Treatment | 0.076 | 0.115 | **0.458*** |
|  |  |  |  |  |
| ∆ Total leukocyte count | Placebo | **0.406*** | 0.306 | 0.332 |
|  | Treatment | -0.009 | 0.115 | **0.458*** |
|  |  |  |  |  |
| ∆ Neutrophils (10^9^/L) | Placebo | **0.462**** | **0.426*** | 0.338 |
|  | Treatment | -0.027 | 0.197 | 0.003 |
|  |  |  |  |  |
| ∆ Monocytes | Placebo | 0.206 | 0.073 | **0.389*** |
|  | Treatment | -0.051 | -0.149 | 0.203 |
|  |  |  |  |  |
| ∆ Lymphocytes | Placebo | -0.100 | -0.267 | 0.031 |
|  | Treatment | 0.067 | 0.038 | **-0.401*** |

Abbreviations: IL: interleukin; R: receptor; s: soluble

Numbers in bold indicate statistical significance at a level of *p < 0.05, **p< 0.01, ***p< 0.001
